# Can virtual reality simulators be a certification tool for bariatric surgeons?

**DOI:** 10.1007/s00464-013-3179-x

**Published:** 2013-08-31

**Authors:** Domenico Giannotti, Gregorio Patrizi, Giovanni Casella, Giorgio Di Rocco, Massimiliano Marchetti, Francesca Frezzotti, Maria Giulia Bernieri, Anna Rita Vestri, Adriano Redler

**Affiliations:** 1 Department of Surgical Sciences, Policlinico “Umberto I”, “Sapienza” – University of Rome, Viale Regina Elena 324, 00161 Rome, Italy; 2 Department of Radiology, Oncology and Anatomy Pathology, “Sapienza” – University of Rome, Rome, Italy; 3 Department of Public Health and Infectious Disease, “Sapienza” – University of Rome, Rome, Italy

**Keywords:** Virtual reality simulators, Bariatric surgery, Gastric bypass, Certification, Laparoscopy

## Abstract

**Background:**

Construct validity of virtual laparoscopic simulators for basic laparoscopic skills has been proposed; however, it is not yet clear whether the simulators can identify the actual experience of surgeons in more complex procedures such as laparoscopic Roux-en-Y gastric bypass. This study tested the ability of the Lap Mentor simulator to recognize the experience in advanced laparoscopic procedures and to assess its role in the certification of bariatric surgeons.

**Methods:**

Twenty surgeons were divided into two groups according to their experience in laparoscopic and bariatric surgery. The general group included 10 general surgeons performing between 75 and 100 nonbariatric laparoscopic procedures. The bariatric group included 10 bariatric surgeons performing between 50 and 100 laparoscopic bariatric procedures. Participants were tested on the simulator in one basic task (task 1: eye–hand coordination) and in two tasks of the gastric bypass module (task 2: creation of the gastric pouch; task 3: gastrojejunal anastomosis).

**Results:**

Comparing the groups, no significant differences were found in task 1. Analyzing the results from the gastric bypass module (bariatric vs. general), in task 2, significant differences (*p* < 0.05) were found in the median volume of the gastric pouch (21 vs. 48 cm^3^), in the percentage of fundus included in the pouch (8.4 vs. 29.4 %), in the complete dissection at the angle of His (10 vs. 3), and in safety parameters. In task 3, significant differences were found in the size and position of enterotomies.

**Conclusions:**

The Lap Mentor may be proposed as a certification tool for bariatric surgeons because it also recognizes their specific skills in the technical details of the procedure that affect long-term results. Furthermore, the possibility of analyzing the performance in detail can help define areas where the surgeon is lacking. These findings indicate a potential role of the Lap Mentor in tailoring the training to maximize improvement.

Over the past two decades, the prevalence of morbid obesity has increased globally. In the United States, obesity is the most frequent chronic disease, affecting more than a third of adults without significant differences in prevalence between men and women at any age [1]. Bariatric surgery has proven to be a successful method for sustained weight loss in morbidly obese patients, and laparoscopic Roux-en-Y gastric bypass (LRYGBP) has become the most commonly performed procedure [2]. LRYGBP is a technically challenging procedure requiring advanced skills such as complex anastomosis creation, bowel manipulation, laparoscopic suturing, and dissection. The learning curve reported in the literature ranges from 50 to 100 cases, and an increased incidence of complications is recorded in this period [3].

The need for specific technical skills of such laparoscopic procedures, time limitations, and legal issues have stimulated the development of training programs outside of the operating theater using virtual reality laparoscopic simulators (VRLS) [4, 5]. VRLS has proven to be a safe and effective training tool to approach laparoscopic surgery, and the development of simulator basic-skills curricula is evolving [6]. Validation of VRLS requires the evidence of five types of validity: content, face, construct, concurrent, and predictive [7]. Construct validity is essential to define the effectiveness of VRLS for training and certification because it demonstrates the ability of a simulator to discriminate between expert and novice surgeons. Several authors have proved construct validity by detecting statistically significant differences in performances measured between subjects with different levels of laparoscopic experience [8–14]. Although this is widely demonstrated for basic laparoscopic skills, there is little evidence for more complex procedures such as LRYGBP [15].

As a result of the growing dissemination of bariatric surgery and increased patient demands, more and more surgeons, even without a specific training, have begun performing bariatric advanced laparoscopic surgical procedures. These procedures require well-defined technical skills and specific knowledge of the pathological mechanisms of disease that can influence both perioperative and long-term outcomes.

Our study aimed to test the ability of the Lap Mentor simulator to recognize the different levels of expertise in advanced laparoscopic procedures, particularly in LRYGBP, and to assess its role in the certification of bariatric surgeons.

## Materials and methods

The study was performed in the Department of Surgical Sciences at “Sapienza”—University of Rome, Italy. The study was approved by the local ethics committee (protocol 518/13). All subjects were enrolled into the study on a voluntary basis, and each participant provided full informed consent.

Before enrollment, all participants completed a questionnaire assessing demographics as well as number and type of previous laparoscopic procedures.

We recruited a total of 20 surgeons and divided them into 2 groups on the basis of their experience in laparoscopic and bariatric surgery. The first group, the bariatric group, included 10 surgeons (mean age 36.7 ± 3.3 years) performing between 50 and 100 laparoscopic bariatric procedures (laparoscopic sleeve gastrectomy, LRYGBP, and adjustable gastric banding) and trained in a dedicated center for bariatric surgery. The second group, the general group, included 10 general surgeons (mean age 37.7 ± 5.8 years) performing from 75 to 100 nonbariatric laparoscopic procedures (cholecystectomy, appendectomy, inguinal and incisional hernia repair, and colectomy).

The number of procedures performed by each surgeon of bariatric group was set at 50–100 procedures, which is the literature reference for the LRYGBP learning curve [3]. In the general group, the selection of 75 or more procedures was chosen to define young general surgeons trained in laparoscopy who have likely reached the plateau of the learning curve in most general surgical laparoscopic procedures. Another inclusion criteria was lack of laparoscopic simulator experience.

All participants were tested on the virtual laparoscopic simulator Lap Mentor in one basic skills task (task 1: eye–hand coordination) and in two tasks of the gastric bypass module (task 2: creation of the gastric pouch; task 3: creation of gastrojejunal anastomosis with linear stapler). Before performing the tasks, each participant viewed a standardized screen, provided by the simulator, in which the procedure was explained while a full intraoperative video illustrating the creation of the stapled gastric pouch and gastrojejunal anastomosis was played.

For each task, we analyzed specific parameters measured and reported by the simulator software to evaluate subjects’ performances. For task 1 (eye–hand coordination), participants located ten flashing blue and red balls and touched them with the tool of the same color. We recorded the total time in seconds to complete the procedure; the accuracy rate was calculated by dividing the number of correct hits by the total number of touched balls. We also recorded the economy of movement of right and left instruments, measured as a percentage and calculated by dividing the ideal path length by the relevant path length of right or left instrument.

In task 2 (creation of the gastric pouch), we recorded the total time in seconds to complete the procedure; the volume of the gastric pouch in cubic centimeters; the percentage of fundus included in the pouch; the percentage of unsafe dissection, calculated by dividing the number of dissection maneuvers performed at a distance of more than 10 mm from the stomach wall by the total number of dissection maneuvers; the time in seconds in which coagulation was unsafely used; the number of serious complications, bleeding incidents, and noncauterized bleeding; the distance in millimeters of the first stomach dissection from gastroesophageal junction; and the number of times the linear cutter was fired. Furthermore, the simulator evaluated whether dissection was performed at the angle of His when at least 50 % of the fat was resected at the left crural area of the diaphragm, and whether the gastric pouch was totally separated from the stomach.

In task 3 (gastrojejunal anastomosis), we recorded the total time needed to achieve the procedure; the number of injuries resulting from jejunal overstretch; the number of punctures larger than 1 cm; the number of punctures not used for the gastrojejunal anastomosis; and the distance of the puncture created on the jejunum from the end of the cut limb.

Continuous variables are presented as mean ± standard deviation. We assessed the normality of the data with the Shapiro–Wilk test. Data of performance metrics do not follow a normal distribution and therefore were reported as median and interquartile range. Categorical variables are presented as counts or percentage. To evaluate the homogeneity and the differences between groups, we used the Mann–Whitney test; a probability value of <0.05 was considered statistically significant. All analyses were carried out by SPSS software version 18.0 (SPSS, Chicago, IL, USA).

## Results

### Task 1: eye–hand coordination

No significant differences were found between the bariatric and general groups, confirming the homogeneity of the two groups for basic laparoscopic skills (Table 1).

**Table 1:** Task 1 (eye–hand coordination) performance metrics

Performance metric	General group		Bariatric group		*p*
	Median	IQR	Median	IQR	
Total time (s)	53.5	41.7–55.2	52.5	34.7–60.2	0.8498
Accuracy rate (%)	84.6	69.3–90.0	84.1	72.9–89.9	0.7050
EMRI	67.2	59.0–70.6	66.3	55.4–69.6	0.5453
EMLI	67.4	54.6–75.6	66.1	57.0–71.4	0.8205

*IQR* interquartile range, *EMRI* economy of movement of right instrument, *EMLI* economy of movement of left instrument

### Task 2: creation of gastric pouch

When we compared the bariatric and general groups, we found significant differences in the volume of the gastric pouch created (median 22.1 vs. 48.3 cm^3^; *p* = 0.0034), in the percentage of fundus included in the pouch (median 8.4 vs. 29.4 %; *p* = 0.0034), and in the distance of the starting point of stomach dissection from gastroesophageal junction (median 47.5 vs. 26.6 mm; *p* = 0.0284).

In the bariatric group, the dissection at the angle of His was performed by all participants compared to three dissections performed in the general group (*p* = 0.0014).

Considering the safety parameters, the time in which coagulation was unsafely used was significantly lower for the bariatric group (median 3.5 vs. 26.5 s; *p* = 0.0006), as was the number of bleeding incidents (median 0 vs. 5.5; *p* = 0.0003) and the number of noncauterized bleeding incidents (median 0 vs. 1; *p* = 0.0006).

No significant differences were found in the other performance metrics recorded (Table 2).

**Table 2:** Task 2 (creation of the gastric pouch) performance metrics

Performance metric	General group		Bariatric group		*p*
	Median	IQR	Median	IQR	
Total time (s)	901.5	711.2–1,161.5	820.0	606.7–1,443.5	0.7913
Pouch volume (cm^3^)					
Unsafe dissection (%)	47.2	39.2–63.8	51.0	40.8–59.5	0.9397
Distance from GE junction (mm)	26.6	23.3–39.0	47.5	36.3–52.4	0.0284
Times the linear cutter was fired (*n*)	3.5	2.7–5.0	3.0	3.0–4.0	0.5408
Fundus included in the pouch (%)	29.4	18.8–42.2	8.4	2.9–14.9	0.0034
Time of unsafe coagulation (s)	26.5	14.5–43.7	3.5	2.0–10.7	0.0006
Complications (*n*)	0.0	0.0–0.2	0.0	0.0–0.0	0.1462
Bleeding (*n*)	5.5	2.0–8.0	0.0	0.0–1.0	0.0003
Noncauterized bleeding (*n*)	1.0	1.0–1.2	0.0	0.0–0.0	0.0006

*IQR* interquartile range, *GE* gastroesophageal

### Task 3: gastrojejunal anastomosis

When comparing the bariatric and general groups, we noticed significant differences in the number of punctures larger than 1 cm (median 0 vs. 1; *p* = 0.0285) and in the distance of the puncture created on the jejunum from the end of the cut limb (median 53.3 vs. 65.8 mm; *p* = 0.0015).

No significant differences were recorded in the total time needed to complete the procedure, in the number of injuries resulting from jejunal overstretch, or in the number of punctures not used for the gastrojejunal anastomosis (Table 3).

**Table 3:** Task 3 (gastrojejunal anastomosis) performance metrics

Performance metric	General group		Bariatric group		*p*
	Median	IQR	Median	IQR	
Total time (s)	306.0	265.7–518.2	385.5	291.5–454.0	0.8501
Jejunum injuries (*n*)	3.5	0.7–7.5	5.5	2.7–7.2	0.3053
Punctures >1 cm (*n*)	1.0	0.0–1.0	0.0	0.0–0.2	0.0285
Distance from jejunum cutter limb (mm)	65.8	61.6–79.4	53.3	51.3–59.9	0.0015
Punctures not used (*n*)	0.0	0.0–0.2	0.0	0.0–0.0	0.1462

*IQR* interquartile range

## Discussion

VRLS provide a measurable objective evaluation that eliminates observer bias because all performance metrics are analyzed and translated into scores by a validated preset software. Assessment of VRLS requires the evidence of five types of validity: content, face, construct, concurrent, and predictive. Content and face validity are subjective qualities that depend on the judgment of the observer, whereas construct, concurrent, and predictive validity provide an objective evaluation based on quantitative measures [7].

For the predictive validity of most commercially available VRLS, the transfer effect of acquired skills to the operative room has been demonstrated [16, 17], as has their concurrent validity by comparing VRLS to other established forms of laparoscopic assessment such as box trainers [18, 19].

Construct validity is achieved when the simulator can discriminate experienced from inexperienced surgeons according to their performance score. Several authors demonstrated construct validity for basic laparoscopic skills, usually by comparing performances of medical students, residents, and surgeons with different experience levels [8–14].

It is not yet clear whether the simulators can identify the actual experience of the surgeon in more complex procedures. This assessment could play a central role in credentialing surgeons for specific procedures and in maintaining certification. Virtual reality simulation currently represents the best way to propose new tools for surgical education and might lead to new frontiers of certification of surgical ability, as is already routinely done for aviation [20]. Several attempts have been made to objectively evaluate surgical performance. In 1997, Reznick et al. [21] modified the objective structured clinical examination (OSCE) and developed the objective structured assessment of technical skills (OSATS) using a global rating scale consisting of seven evaluation items scored on a 5-point scale: respect for tissue, time/motion, instrument handling, flow of operation, knowledge of instruments, knowledge of procedure, and use of assistants.

In 2006, Matsuda et al. [22] developed a system for reviewing unedited videotapes of laparoscopic nephrectomies or adrenalectomies using simplified criteria to assess the laparoscopic surgical skills of urologists.

Lewis et al. [15] first attempted to demonstrate construct and concurrent validity of a VRLS for advanced laparoscopic training, recruiting 20 surgeons of varying experience (10 novice, 5 intermediate, 5 experienced) to perform a jejunojejunostomy on both cadaveric tissue and on the bariatric module of the Lap Mentor simulator. Construct validity was achieved by assessing videos of virtual reality simulated jejunojejunostomy performed by the different groups according to OSATS global rating scales. Evaluating unedited surgical videos by two blinded experts according to OSATS or similar criteria [23] currently appears to be a valid and reliable system of proficiency assessment [24].

However, some biases can affect the assessment process as a result of the subjective judgment of observers and a decrease in their attention level, especially in longer procedures. Our study suggests that the gastric bypass module of Lap Mentor provides objective measurements of surgical skills with a future potential role in the certification of dedicated laparoscopic surgeons.

When comparing the bariatric and general groups, we did not find significant differences in the eye–hand coordination task (task 1), according to the homogeneity of the two groups for laparoscopic basic skills (Table 1). Unexpectedly, no significant differences (*p* < 0.05) were found in the creation of gastrojejunal anastomosis (task 3), except for the smaller size of enterotomies performed by bariatric surgeons and for the minor distance of the puncture created on the jejunum from the end of the cut limb (median 53.3 vs. 65.8 mm; *p* = 0.0015). A possible explanation is that enteric anastomoses are not exclusively performed in bariatric surgery; general surgeons were also experienced in these procedures (Table 3). For the creation of the gastric pouch (task 2), when we compared the two groups, we found significant differences in several performance metrics and technical details, with possible implications in terms of perioperative and long-term outcomes of the procedure (Table 2).

**Fig. 1:**
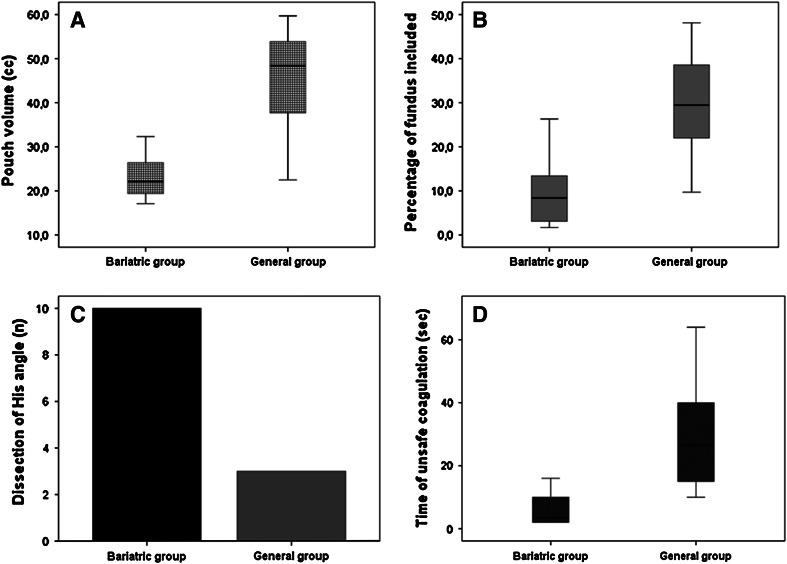
Comparison of the results of the 2 groups for **A** the volume of the gastric pouch in cubic centimeters, **B** the percentage of fundus included in the pouch, **C** the number of surgeons in each group who completed dissection of the angle of His, and **D** the time in seconds of unsafe coagulation during the procedures

Bariatric surgeons created gastric pouches of smaller volume (median 22.1 vs. 48.3 cm^3^; *p* = 0.0034), including lesser percentage of fundus (median 8.4 vs. 29.4 %; *p* = 0.0034), and completing in all cases the dissection at the angle of His (Fig. 1A–C).

It is surprising to note that in task 2, the bariatric group started dissection further from the gastroesophageal junction compared to the general group, even if the pouch was smaller for bariatric surgeons. The bariatric group probably followed simulator instructions more strictly and started dissection between the second and third vessels on the lesser curve of the stomach, while the general group started dissection higher on the curve. Despite this difference in length from the junction, bariatric surgeons created a smaller pouch by completely resecting the fundus.

Clinical evidence has demonstrated that the volume of the pouch affects weight loss [25]. Such a parameter is so relevant that in cases of failure of LRYGBP, resizing the pouch is considered a possible option in revisional surgery [26]. Moreover, as reported in detail in the results of our study, the better performance of the bariatric group in safety parameters demonstrated the ability of the Lap Mentor simulator to identify the technical skills of surgeons and their specific knowledge in vascular anatomy (Fig. 1D). Bariatric surgeons may have acquired more confidence with the anatomy of gastroesophageal district because all bariatric procedures (gastric banding, sleeve gastrectomy, and LRYGBP) are related to the stomach.

Our study should be interpreted in the context of several limitations. First, because bariatric surgery is rather recent, there are few dedicated surgeons. This led to a limited sample size: we recruited as many bariatric surgeons as met our inclusion criteria, and we reached only ten physicians. For this reason, we have chosen to compare them only with ten general surgeons. This is basically a convenience sample. Moreover, because our study aims to prove the validity of simulators as a certification tool, we enrolled surgeons who had just come out of the learning curve; they are the real target of recruiting structures.

Second, we conducted a single session of tests. This limit was chosen to avoid the familiarization effect that we met in previous studies [27]. Familiarization is observed when participants practice more than once on a given device. In the first approach, subjects get to know the device; in the second or third test, they are already familiar to it and achieve better results [28]. This would have led to adaptive biases of the single participants linked to their personal surgical experience. Therefore, to minimize this effect, we had the choice between two options: we could either schedule a single session, or we could arbitrarily schedule a variable number of sessions for each laparoscopist according to the estimated experience of the surgeon, which would have led to a loss of objectivity of the study.

Third, this study lacked verification in real surgical procedures. It would have been interesting to test all surgeons on such procedures in the operating room and to score them with OSATS to evaluate the translation of skills from simulator to surgery. Such a test would be hard to justify on ethical and/or legal grounds.

Simulators provide objective measures of a surgeon’s technical skills in laparoscopy that can be further associated with OSATS scales during surgical procedures. We demonstrated construct validity of the Lap Mentor bariatric module, therefore suggesting that simulators can be easily used for training and certification of laparoscopic surgeons even in advanced laparoscopic procedures. Furthermore, the possibility of analyzing the performance in detail can help define areas where the surgeon is lacking. These findings also demonstrate a potential role in tailoring training on the tasks that can help to maximize improvement. Finally, our experiment shows that simulators might be a useful tool in recruitment of new surgeons by evaluating them for expertise required in specific fields.
